# Assessment of distribution center locations using a multi-expert subjective–objective decision-making approach

**DOI:** 10.1038/s41598-021-98698-y

**Published:** 2021-09-30

**Authors:** Mehdi Keshavarz-Ghorabaee

**Affiliations:** Department of Management, Faculty of Humanities (Azadshahr Branch), Gonbad Kavous University, 49717-99151 Gonbad Kavous, Iran

**Keywords:** Information technology, Engineering

## Abstract

Distribution is a strategic function of logistics in different companies. Establishing distribution centers (DCs) in appropriate locations helps companies to reach long-term goals and have better relations with their customers. Assessment of possible locations for opening new DCs can be considered as an MCDM (Multi-Criteria Decision-Making) problem. In this study, a decision-making approach is proposed to assess DC locations. The proposed approach is based on Stepwise Weight Assessment Ratio Analysis II (SWARA II), Method based on the Removal Effects of Criteria (MEREC), Weighted Aggregated Sum Product Assessment (WASPAS), simulation, and the assignment model. The assessment process is performed using the subjective and objective criteria weights determined based on multiple experts’ judgments. The decision matrix, subjective weights and objective weights are modeled based on the triangular probability distribution to assess the possible alternatives. Then, using simulation and the assignment model, the final aggregated results are determined. A case of DC locations assessment is addressed to show the applicability of the proposed approach. A comparative analysis is also made to verify the results. The analyses of this study show that the proposed approach is efficient in dealing with the assessment of DC locations, and the final results are congruent with those of existing MCDM methods.

## Introduction

Distribution can be considered a key driver of the overall profitability of a company since both customer experience and supply chain costs are affected by it. A distribution center (DC) can help a company for delivering its products to customers. In modern supply chains, distribution centers play an essential role in logistics and could affect the company’s performance^1–3^. The time of storage and operation is the main difference between a distribution center and a warehouse. In a distribution center, the time between receiving is faster, but in the case of the warehouse, this time is longer^4^. However, in some references, distribution centers have been categorized as a type of warehouses^5^. Bancroft^6^ studied the strategic role of the distribution centers. He stated that warehousing is transportation with zero velocity, and it doesn’t add any value to the product stored in a warehouse, but on the other hand distribution is a dynamic process that moves the goods from the origin to the customer with a fairly continuous flow. Although both warehousing and distribution need keeping inventory in the supply chain, the amount of this inventory and the time of being stationary make them significantly different^6^. Due to the importance of distribution in different types of supply chains, several problems related to this activity have been addressed in the literature. Inventory control^7,8^, optimal bundling^9^, sustainability^10,11^, network optimization^12^, vehicle routing^13^, and scheduling^14^ are some of the problems which have been studied by researchers. In addition to these problems, location assessment is a fundamental problem for different elements in any type of supply chain since it can make an efficient flow of products and information from the upstream part of the supply chain (in particular procurement and production) to the downstream part of it (in particular retailers and customers). The location assessment problem is also a vital problem in other fields like construction management^15^, medical service management^16^, waste management^17^, energy management^18^, and so on.

To start the location assessment process for establishing a new distribution center, companies may have different reasons. Expansion of capacity to help business growth, cost reduction, competition in new markets, rationalization after an acquisition or merger, tapping into new labor pools, and dealing with geopolitical developments are some of the possible reasons^19–21^. The establishment of a new distribution center requires significant investments (or leases). The investment decisions on opening new distribution centers are long-term decisions that may lead to a long-term impact on different processes of a company. Actually, if a company makes a wrong decision on the location of new distribution centers, there will be a negative impact for a prolonged period (e.g., higher costs, customs issues, issues in finding the proper labor, delivery issues)^22–24^. Therefore, establishing distribution centers in suitable locations is a vital decision because of its costs, irreversible nature of the decision, and long-term commitments^25^. Assessment of the possible places or sites for locating distribution centers can be considered as a multi-criteria decision-making (MCDM) problem^26^. Sometimes, mathematical models are used to determine the optimal locations of the distribution centers based on different parameters like distance and expected demand^27^, but in several cases, expert-based decisions are more suitable to assess the possible sites^28^.

The focus of this study is on an expert-based assessment in the process of selecting appropriate sites for distribution centers. When we have more than one expert in an assessment process, we need to use an approach to ensure that the experts’ judgments and opinions are covered well. There have been several decision-making approaches used to handle experts’ judgments and opinions^29–34^. Group decision-making approaches, which are based on multiple experts, have widely been used in different fields of study, including engineering, business, biology, economics, and so on^35–37^. When we use an expert-based decision-making approach, we need to involve the subjective importance of criteria expressed by the experts or decision-makers as well as the objective importance of criteria, which can be determined according to the decision matrix. The decision matrix itself could be constructed based on the judgments of the experts, the objective evaluation of alternatives, or a combination of them. To have more reliable results in an MCDM problem with multiple experts, we need to be able to consider the subjective and objective importance or weights of criteria^38,39^.

There have been several studies on the assessment and selection of distribution centers using MCDM approaches. In Table 1, a summary of recent studies and some of their features are presented, and the current study is also characterized. The MCDM approaches used in these studies include Decision Making Trial and Evaluation Laboratory (DEMATEL), Technique for Order of Preference by Similarity to Ideal Solution (TOPSIS), Grey Relational Analysis (GRA), Multi-Objective Optimization on the basis of Ratio Analysis (MOORA), ELimination Et Choix Traduisant la REalité (ELECTRE), Operational Competitiveness Rating Analysis (OCRA), Analytic Hierarchy Process (AHP), Analytic Network Process (ANP), Preference Ranking Organization Method for Enrichment of Evaluations (PROMETHEE), Weighted Aggregated Sum Product Assessment (WASPAS), VIseKriterijumska Optimizacija I Kompromisno Resenje (VIKOR), Combined Compromise Solutions (CoCoSo), Method based on the Removal Effects of Criteria (MEREC) and Stepwise Weight Assessment Ratio Analysis (SWARA).

**Table 1:** Recent studies on the assessment of distribution centers.

No	Year	Author(s)	Approach	Subjective weighting	Objective weighting	Multi-expert
1	2011	Amiri, et al.^40^	BSC (Balanced Scorecard) and DEMATEL	✓	✕	✕
2	2012	Jia and Yang^41^	Entropy and TOPSIS	✕	✓	✕
3	2013	Chakrabort et al.^42^	GRA, MOORA, ELECTRE and OCRA	✕	✕	✕
4	2013	Bouhana et al.^43^	Fuzzy preferences	✓	✕	✓
5	2014	Jin and Yan^44^	Fuzzy AHP	✓	✕	✕
6	2014	Biswas and Hasan^45^	Fuzzy preferences	✓	✕	✕
7	2014	Bagum and Rashed^46^	AHP	✓	✕	✕
8	2015	Dobrota et al.^28^	Fuzzy AHP	✓	✕	✕
9	2015	Simić et al.^47^	AHP and PROMETHEE	✓	✕	✕
10	2016	Agrebi et al.^48^	ELECTRE	✓	✕	✓
11	2016	Cheng and Zhou^49^	Fuzzy AHP	✓	✕	✕
12	2017	Agrebi et al.^50^	ELECTRE	✓	✕	✓
13	2017	Ahmed et al.^51^	AHP and Adaptive Neuro-Fuzzy Inference System	✓	✕	✕
14	2017	Sun et al.^52^	AHP and Linear Programming	✓	✕	✕
15	2017	Tuan and Hien^53^	Fuzzy TOPSIS	✓	✕	✓
16	2018	Li et al.^54^	Entropy and TOPSIS	✕	✓	✕
17	2019	Li and Wang^55^	AHP	✓	✕	✕
18	2019	Ocampo et al.^56^	DEMATEL, ANP and AHP	✓	✕	✕
19	2019	Titiyal et al.^57^	DEMATEL, ANP and VIKOR	✓	✕	✕
20	2019	Mihajlović et al.^58^	AHP and WASPAS	✓	✕	✕
21	2020	Liu and Li^59^	2-dimensional linguistic	✓	✕	✕
22	2020	Yılmaz and Kabak^60^	AHP, TOPSIS, and Interval type-2 fuzzy sets	✓	✕	✓
23	2020	Agrebi and Abed^61^	Fuzzy ELECTRE	✓	✕	✓
24	2020	Liao et al.^62^	CoCoSo and Pythagorean fuzzy sets	✓	✕	✓
25	2021	Kieu et al.^63^	AHP, CoCoSo, and Spherical fuzzy sets	✓	✕	✕
Current study			SWARA, MEREC, WASPAS, Simulation, and Assignment Model	✓	✓	✓

According to Table 1, subjective weighting, objective weighting, and decision-making with multiple experts have not been taken into account concurrently in several studies on distribution centers assessment. Although the assessment approaches have been evolved during the past years, it can be said that we still need to develop and extend more MCDM approaches that are able to quantify the objective and subjective weights simultaneously. The main aim of this study is to propose such an approach to assess different location alternatives for establishing a distribution center based on the opinions and judgments of a group of experts. Moreover, a subsidiary aim is to modify the SWARA method and introduce an improved version of it.

In this paper, a new multi-expert subjective–objective decision-making approach is proposed to deal with such MCDM problems. The proposed approach is an integration of a modified version of SWARA as a subjective weighting method^64^, MEREC as an objective weighting approach^65^, the Monte Carlo simulation as a modeling tool, WASPAS as an MCDM method^66^, and the mathematical assignment model as an aggregation tool. A modified version of the SWARA, called SWARA II, is introduced in this study as a new simple and efficient subjective weighting method. A lower number of comparisons and simplicity are two essential benefits of the SWARA method compared with the other subjective weighting methods. Besides, MEREC uses an exclusion perspective instead of the inclusion perspective, which is the foundation of the other objective weighting methods, to obtain objective criteria weights. Integrating MEREC and SWARA II together provides us with a new perspective in determining the objective weights and a simple way of computing the subjective weights. The triangular distribution is used to model experts’ opinions related to decision matrix, subjective, and objective criteria weights. Then based on the Monte Carlo simulation, a set of the subjective weights determined by SWARA II and a set of objective weights calculated using MEREC are combined, and the WASPAS method is used to compute a score for each alternative in each rank. After making the simulation for a specific number of iterations, the normalized scores are calculated, and the assignment model is solved to perform the final assessment of the alternatives. The proposed approach is applied to a real-world problem to assess distribution centers locations. The results of the proposed approach are finally compared with those of some existing MCDM methods.

## Methodology

The methodology of this study consists of different elements. The main elements of the approach are SWARA II, MEREC, WASPAS, Monte Carlo simulation, and an assignment model. The following subsections delineate these elements, and in the final subsection, we describe the procedure of the proposed approach. Since the proposed approach is applicable in dealing with MCDM problems, in all parts of this section, it is supposed that we have an MCDM problem with $n$ alternatives and $m$ criteria, and the general decision matrix is defined as follows.

1 $$X = \left[ {\begin{array}{*{20}l} {\begin{array}{*{20}c} {x_{11} } \\ {x_{21} } \\ {\begin{array}{*{20}c} \vdots \\ {x_{i1} } \\ {\begin{array}{*{20}c} \vdots \\ {x_{n1} } \\ \end{array} } \\ \end{array} } \\ \end{array} } \hfill & {\begin{array}{*{20}c} {x_{12} } \\ {x_{22} } \\ {\begin{array}{*{20}c} \vdots \\ {x_{i2} } \\ {\begin{array}{*{20}c} \vdots \\ {x_{n2} } \\ \end{array} } \\ \end{array} } \\ \end{array} } \hfill & {\begin{array}{*{20}c} {\begin{array}{*{20}c} \cdots \\ \cdots \\ {\begin{array}{*{20}c} \ddots \\ \cdots \\ {\begin{array}{*{20}c} \ddots \\ \cdots \\ \end{array} } \\ \end{array} } \\ \end{array} } & {\begin{array}{*{20}c} {x_{1j} } \\ {x_{2j} } \\ {\begin{array}{*{20}c} \vdots \\ {x_{ij} } \\ {\begin{array}{*{20}c} \vdots \\ {x_{nj} } \\ \end{array} } \\ \end{array} } \\ \end{array} } & {\begin{array}{*{20}c} {\begin{array}{*{20}c} \cdots \\ \cdots \\ {\begin{array}{*{20}c} \ddots \\ \cdots \\ {\begin{array}{*{20}c} \ddots \\ \cdots \\ \end{array} } \\ \end{array} } \\ \end{array} } & {\begin{array}{*{20}c} {x_{1m} } \\ {x_{2m} } \\ {\begin{array}{*{20}c} \vdots \\ {x_{im} } \\ {\begin{array}{*{20}c} \vdots \\ {x_{nm} } \\ \end{array} } \\ \end{array} } \\ \end{array} } \\ \end{array} } \\ \end{array} } \hfill \\ \end{array} } \right]$$

### SWARA II

There are several methods to determine subjective criteria weights such as AHP, ANP, Best–Worst Method (BWM), Full Consistency Method (FUCOM), and Level Based Weight Assessment (LBWA)^67–70^. In this section, a modified version of the SWARA method is presented. A lower number of comparisons and simplicity are some of the benefits of SWARA over the other methods^64^. Since the overall structure of this modified version is the same as the original method, we called it SWARA II. Like the SWARA method, SWARA II uses a procedure that involves sorting and preferences of criteria. However, some modifications in the procedure make SWARA II easier and more practical for decision-makers to use. The steps of SWARA II to determine subjective criteria weights are as follows.

*Step 1.* Sort the criteria in descending order of importance, i.e., the first criterion in the sorted list has the highest importance. Let us denote by $t_{j}$ the position or rank of the $j$ th criterion in the sorted list ($t_{j} \in \left\{ {1,2, \ldots ,m} \right\}$).

*Step 2.* Ask the decision-maker to express the relative preference ($RP$) concerned with each criterion by comparing it with the next criterion in the sorted list of Step 1. The following question could be used to elicit the preferences of the decision-maker.

“How much more important is the $\left[ {t_{j} } \right]$ th criterion than the $\left[ {t_{j} + 1} \right]$ th criterion?”

Linguistic variables and the Likert scale can be used to answer this question. In this study, we use linguistic variables and their corresponding values defined in Table 2.

**Table 2:** Linguistic variables and their corresponding values.

Linguistic variable	Value
VVL (Very Very Low)	1
VL (Very Low)	2
L (Low)	3
ML (Medium–Low)	4
M (Medium)	5
MH (Medium–High)	6
H (High)	7
VH (Very High)	8
VVH (Very Very High)	9

*Step 3.* Determine the preference degree ($PD$) of each criterion. To determine the values of $PD$ we need to quantify the relative preferences of Step 2 first. If the quantified value of the relative preference of the $\left[ {t_{j} } \right]$ th criterion is denoted by $P_{{\left[ {t_{j} } \right]}}$, we can define the values of $PD$ as follows.

2 $$PD_{{\left[ {t_{j} } \right]}} = u\left( {P_{{\left[ {t_{j} } \right]}} } \right)$$

$u$ is a utility function that turns the quantified values of the relative preferences into some scaled values in the range [0,1], and therefore $0 \le PD_{{\left[ {t_{j} } \right]}} \le 1$. In this study, we use Eq. (3) as a nonlinear utility function; nevertheless, this function can be defined according to decision-makers’ opinions and the characteristics of the problem.

3 $$u\left( x \right) = \left( \frac{x}{10} \right)^{2}$$

**Figure 1:**
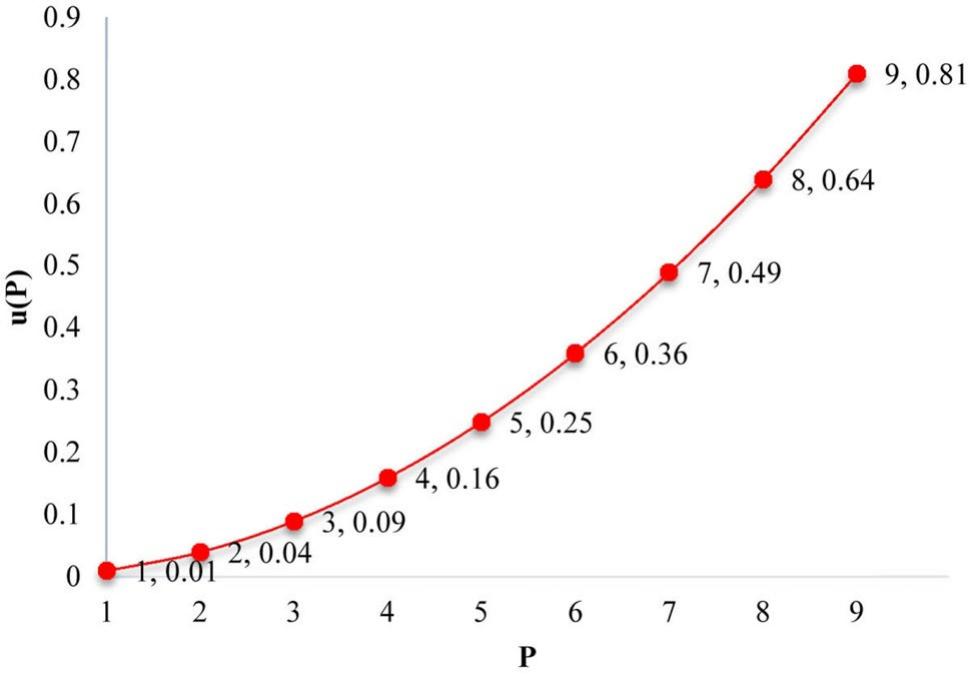
The diagram of the utility function.

According to Table 2, the diagram of the utility function defined in Eq. (3) is shown in Fig. 1.

*Step 4.* Calculate relative weighting coefficients. These coefficients are calculated based on the position of each criterion in the sorted list and the values of $PD$. Let $V_{{\left[ {t_{j} } \right]}}$ denote the values of relative weighting coefficients. Starting from the $m$ th criterion, the following equation is used for the calculation.

4 $$V_{{\left[ {t_{j} - 1} \right]}} = \left( {1 + PD_{{\left[ {t_{j} - 1} \right]}} } \right) \times V_{{\left[ {t_{j} } \right]}}$$

where $$1 \le V_{{\left[ {t_{j} } \right]}} \le 2$$ and $$V_{m} = 1$$.

*Step 5.* Determine the subjective weights of criteria. The subjective weights are determined by scaling the values of relative weighting coefficients. Equation (5) is used in this step.

5 $$w_{j}^{s} = \frac{{V_{{\left[ {t_{j} } \right]}} }}{{\mathop \sum \nolimits_{{t_{j} = 1}}^{m} V_{{\left[ {t_{j} } \right]}} }}$$

### Example

*Steps 1 and 2.* Suppose that we have 4 criteria: $C_{1} , C_{2} , C_{3}$ and $C_{4}$. The criteria are sorted according to the decision-maker’s opinion, and the relative preference concerned with each criterion is given based on the linguistic variables defined in Table 2. The first three columns of Table 3 show the sorted list and relative preferences. As it can be seen, $C_{3}$ is the most important, and $C_{2}$ is the least important criterion.

**Table 3:** The results of the example.

Sorted criteria ($C_{j}$)	$t_{j}$	$RP$	$P_{{\left[ {t_{j} } \right]}}$	$PD_{{\left[ {t_{j} } \right]}}$	$V_{{\left[ {t_{j} } \right]}}$	$w_{j}^{s}$
$C_{3}$	1	VL	2	0.04	1.64	0.29
$C_{4}$	2	ML	4	0.16	1.58	0.28
$C_{1}$	3	MH	6	0.36	1.36	0.24
$C_{2}$	4	–	–	–	1	0.18

*Step 3.* The preference degree of each criterion ($PD_{{\left[ {t_{j} } \right]}}$) is calculated based on the values of $P_{{\left[ {t_{j} } \right]}}$ provided in Table 3 and Eqs. (2) and (3). The results are shown in the fourth and fifth columns of Table 3.

*Step 4.* Relative weighting coefficients are calculated in this step. Since $m = 4$, first, we assign $V_{4} = 1$. Then we can calculate the other coefficients as follows: $V_{3} = \left( {1 + 0.36} \right) \times 1 = 1.36,{ }V_{2} = \left( {1 + 0.16} \right) \times 1.36 = 1.58$ and $V_{1} = \left( {1 + 0.04} \right) \times 1.58 = 1.64$ (Sixth column of Table 3).

*Step 5.* According to the values of $V_{{\left[ {t_{j} } \right]}}$ we will have $\mathop \sum \limits_{{t_{j} = 1}}^{m} V_{{\left[ {t_{j} } \right]}} = 5.58$, so the subjective weights are determined as follows (seventh column of Table 3).

$$\begin{aligned} & w_{1}^{s} = \frac{{V_{{\left[ {t_{1} } \right]}} }}{{\mathop \sum \nolimits_{{t_{j} = 1}}^{4} V_{{\left[ {t_{j} } \right]}} }} = \frac{1.36}{{5.58}} = 0.24 \\ & w_{2}^{s} = \frac{{V_{{\left[ {t_{2} } \right]}} }}{{\mathop \sum \nolimits_{{t_{j} = 1}}^{4} V_{{\left[ {t_{j} } \right]}} }} = \frac{1}{5.58} = 0.18 \\ & w_{3}^{s} = \frac{{V_{{\left[ {t_{3} } \right]}} }}{{\mathop \sum \nolimits_{{t_{j} = 1}}^{4} V_{{\left[ {t_{j} } \right]}} }} = \frac{1.64}{{5.58}} = 0.29 \\ & w_{4}^{s} = \frac{{V_{{\left[ {t_{4} } \right]}} }}{{\mathop \sum \nolimits_{{t_{j} = 1}}^{4} V_{{\left[ {t_{j} } \right]}} }} = \frac{1.58}{{5.58}} = 0.28 \\ \end{aligned}$$

### MEREC

Several methods have been proposed to determine objective criteria weights like Entropy, CRITIC, Standard Deviation (SD), and so on^71–73^. The MEREC method is a new objective weighting method that uses removal effects of criteria in the decision matrix to determine their importance. Unlike the other methods, instead of the inclusion perspective, MEREC focuses on an exclusion perspective and removal effects for the determination of objective criteria weights. The efficiency of this method was demonstrated through simulation-based and comparative analyses. Suppose that we have an MCDM problem involving $n$ alternatives and $m$ criteria^65^. The steps of MEREC are as follows.

*Step 1.* Construct the decision matrix. Suppose that we have a decision matrix like Eq. (1) and $x_{ij} > 0$.

*Step 2.* Normalize the decision matrix and transform all values into the minimization type. $n_{ij}^{x}$ denotes the normalized matrix elements. If ${\text{B}}^{S}$ shows the set of beneficial criteria, and ${\text{C}}^{S}$ represents the set of non-beneficial criteria, we can utilize the following equation for normalization.

6 $$n_{ij}^{x} = \left\{ {\begin{array}{*{20}c} {\frac{{\mathop {\min }\limits_{k} x_{kj} }}{{x_{ij} }} \quad if\quad j \in {\text{B}}^{S} } \\ {\frac{{x_{ij} }}{{\mathop {\max }\limits_{k} x_{kj} }} \quad if\quad j \in {\text{C}}^{S} } \\ \end{array} } \right.$$

*Step 3.* Calculate the performance of the alternatives ($S_{i}$) using a logarithmic measure. We can calculate these values using the following equation.

7 $$S_{i} = ln\left( {1 + \left( {\frac{1}{m}\mathop \sum \limits_{j} \left| {ln\left( {n_{ij}^{x} } \right)} \right|} \right)} \right)$$

*Step 4.* Calculate the performance of the alternatives by removing each criterion. If the performance of $i$ th alternative concerning the removal of the $j$ th criterion is symbolized by $S_{ij}^{^{\prime}}$, Eq. (8) is used to calculate the values of $S_{ij}^{^{\prime}}$.

8 $$S_{ij}^{^{\prime}} = ln\left( {1 + \left( {\frac{1}{m}\mathop \sum \limits_{{k,{ }k \ne j}} \left| {ln\left( {n_{ik}^{x} } \right)} \right|} \right)} \right)$$

*Step 5.* Obtain the removal effect of the $j$ th criterion by computing the summation of absolute deviations related to the values resulted from Steps 3 and 4 of the method. Let us denote by ${\mathcal{E}}_{j}$ the removal effect of the $j$ th criterion. Using the following equation, we can calculate the values of ${\mathcal{E}}_{j}$.

9 $${\mathcal{E}}_{j} = \mathop \sum \limits_{i} \left| {S_{ij}^{^{\prime}} - S_{i} } \right|$$

*Step 6.* Determine the objective weights of criteria using the values of removal effects (${\mathcal{E}}_{j}$) obtained in the previous step. If $w_{j}^{o}$ stands for the objective weight of the $j$ th criterion, we use Eq. (10) for calculating $w_{j}^{o}$.

10 $$w_{j}^{o} = \frac{{{\mathcal{E}}_{j} }}{{\mathop \sum \nolimits_{k} {\mathcal{E}}_{k} }}$$

### WASPAS

The WASPAS method is an efficient MCDM method that has been applied to several real-world problems in different disciplines. The method is actually an integration of two prevalent MCDM methods. weighted sum model (WSM) and weighted product model (WPM). You can see the steps of this method in the following.

*Step 1.* Normalize the decision matrix elements using a linear normalization approach, as follows.

11 $$\overline{x}_{ij} = \left\{ {\begin{array}{*{20}c} {\frac{{x_{ij} }}{{\mathop {\max }\limits_{i} x_{ij} }} if\quad j \in {\text{B}}^{S} } \\ {\frac{{\mathop {\min }\limits_{i} x_{ij} }}{{x_{ij} }} if\quad j \in {\text{C}}^{S} } \\ \end{array} } \right.$$

The sets of beneficial and non-beneficial criteria in Eq. (11) are symbolized by ${\text{B}}^{S}$ and ${\text{C}}^{S}$, respectively.

*Step 2.* Determine the measures of weighted sum model (${\mathcal{M}}_{i}^{S}$) and weighted product model (${\mathcal{M}}_{i}^{P}$) for the set of alternatives ($w_{j}$ denotes the weight of $j$ th criterion).

12 $${\mathcal{M}}_{i}^{S} = \mathop \sum \limits_{j = 1}^{m} w_{j} \overline{x}_{ij}$$

13 $${\mathcal{M}}_{i}^{P} = \mathop \prod \limits_{j = 1}^{m} \left( {\overline{x}_{ij} } \right)^{{w_{j} }}$$

*Step 3.* Calculate the WASPAS measure by combining the values of ${\mathcal{M}}_{i}^{S}$ and ${\mathcal{M}}_{i}^{P}$ method for the set of alternatives as follows.

14 $${\mathcal{M}}_{i} = \vartheta {\mathcal{M}}_{i}^{S} + \left( {1 - \vartheta } \right){\mathcal{M}}_{i}^{P}$$

In the above equation, $\vartheta$ is the combination parameter of the WASPAS method. In this study, the parameter has the value 0.5 ($\vartheta = 0.5$). This value is common in most studies that used the WASPAS method and can make a balance between WSM and WPM measures^64^.

*Step 4.* Evaluate and rank the alternatives in terms of decreasing values of ${\mathcal{M}}_{i} .$

### Monte Carlo simulation

In simple terms, Monte Carlo simulation can be defined as any procedure which involves randomly generated numbers for solving a problem. A problem may have components that can be identified with some random variables which follow probability distributions. To deal with an MCDM problem, we should sometimes consider the opinions of several experts or decision-makers in the assessment process. Using probability distributions to model experts’ opinions could be helpful to handle this type of problem. In this study, we use the triangular distribution as a simple and efficient distribution to model the opinions of a group of experts or decision-makers. This distribution has three parameters, and we approximately define these parameters based on the minimum, maximum, and average values of a sample.

### Assignment model

The assignment model is a classic integer linear programming model with zero–one or binary variables. This model is an instrumental model in dealing with different practical problems. This model is also applicable in MCDM problems. The study made by Bernardo and Blin^74^ is one of the first applications of the assignment model in MCDM problems. The following model describes this model to deal with a decision-making problem.

15 $$\begin{aligned} & Max\;f = \mathop \sum \limits_{i = 1}^{n} \mathop \sum \limits_{r = 1}^{n} \pi_{ir} s_{ir} \\ & {\text{subject}}\;{\text{to}}, \\ & \quad \quad \quad \quad \mathop \sum \limits_{i = 1}^{n} s_{ir} = 1 \quad \forall i \\ & \quad \quad \quad \quad \mathop \sum \limits_{r = 1}^{n} s_{ir} = 1 \quad \forall r \\ & \quad \quad \quad \quad s_{ir} \in \left\{ {0,1} \right\} \\ \end{aligned}$$

In the above-mentioned model, $\pi_{ir}$ is a measure of concordance among all criteria on ranking the $i$ th alternative at the $r$ th place. If the $i$ th alternative is placed at the $r$ th rank, the value of $s_{ir}$ equals 1 ($s_{ir} = 1$).

### Proposed approach

**Figure 2:**
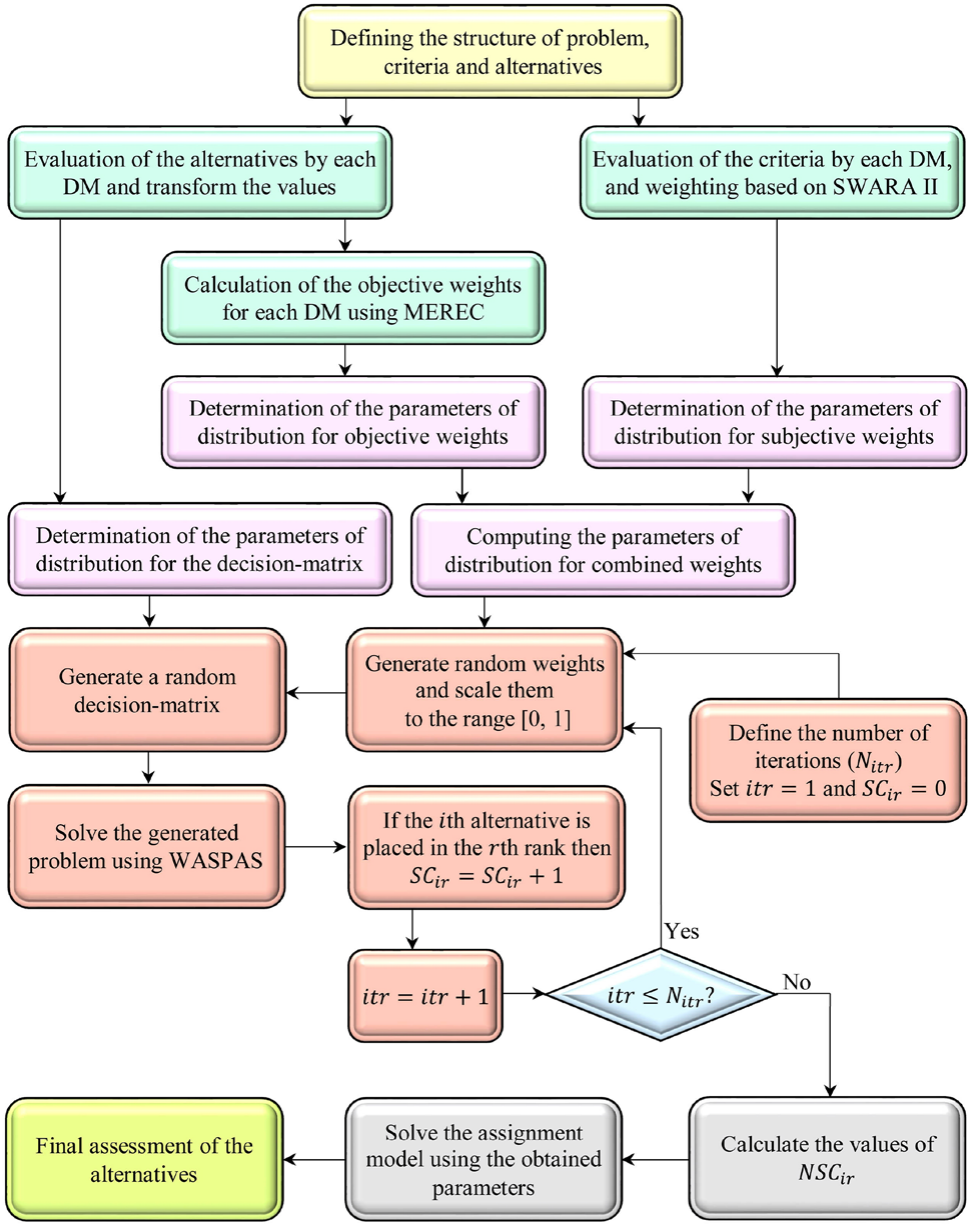
The flowchart of using the proposed approach.

In this section, we describe the procedure of using the proposed approach. Flowchart of the proposed approach is depicted in Fig. 2. The proposed approach can be used in a step-by-step way as shown as follows.

*Step 1.* Define the problem. In this step, the structure of the problem, the alternatives and the criteria should be defined based on experts’ opinions. For this purpose, a group of experts or decision-makers should be formed first. Suppose that we have a group of $d$ experts or decision-makers. An agreement on the alternatives and criteria and their definitions is essential in this step. To reach the agreement, a consensus method like Delphi could be used. Here, it is presumed that there are $n$ alternatives and $m$ criteria.

*Step 2.* Evaluate the importance of criteria. In this step, the SWARA II method (presented in the previous subsection) is used, and the subjective weights of the criteria are determined based on decision-makers’ expressions. Therefore, we have $d$ sets of subjective criteria weights (one set for each decision-maker). Let us denote by $w_{jk}^{s}$ the subjective weight of the $j$ th criterion related to the $k$ th decision-maker.

*Step 3.* Evaluate the performance of the alternatives on each criterion. In this step, the decision-makers are asked to express their opinion on the performance of the alternatives on each criterion. To elicit the decision-makers’ opinions, we can use linguistic variables presented in Table 2. Then these expressions are transformed into numerical values, which are also defined in Table 2. Consequently, we have a decision matrix for each decision-maker. We will denote by $X_{k}$ the decision matrix corresponding to the $k$ th decision-maker, and $x_{ijk}$ shows the rating of the $i$ th alternative on the $j$ th criterion in $X_{k}$.

*Step 4.* Calculate the objective weights. In this section, the MEREC method is used to determine the objective weights of each criterion related to each decision-maker. As previously mentioned, we need to know the decision matrix and the type of criteria to obtain objective weights by MEREC. The decision-matrices related to different decision-makers ($X_{k}$) are used for the calculation of the objective weights. Then we will have a set of objective weights for each decision-maker. Hereafter, $w_{jk}^{o}$ is used to show the objective weight of the $j$ th criterion connected with the $k$ th decision-maker.

*Step 5.* Determine the distribution parameters of the decision matrix. In this step, the triangular distribution is used to model the decision-matrices of different decision-makers. Suppose that $x_{ij}$ represents a random variable for the performance of the $i$ th alternative on the $j$ th criterion, and $x_{ij}^{l}$, $x_{ij}^{m}$, and $x_{ij}^{u}$ denote the lower, middle, and upper parameters for the triangular distribution of $x_{ij}$. Then the parameters can be estimated using the following equations.

16 $$x_{ij}^{l} = \mathop {\min }\limits_{k} x_{ijk}$$

17 $$x_{ij}^{m} = \frac{1}{d}\mathop \sum \limits_{k = 1}^{d} x_{ijk}$$

18 $$x_{ij}^{u} = \mathop {\max }\limits_{k} x_{ijk}$$

*Step 6.* Determine the distribution parameters of subjective weights. In this step, we need to determine three parameters of the triangular distribution for subjective weights obtained using SWARA II in Step 2. Here we define $w_{j}^{s}$ as a random variable for subjective weights of criteria. Let us denote by $w_{j}^{sl}$, $w_{j}^{sm}$, and $w_{j}^{su}$ the lower, middle, and upper parameters of the distribution of $w_{j}^{s}$. The following equations are used to determine these parameters.

19 $$w_{j}^{sl} = \mathop {\min }\limits_{k} w_{jk}^{s}$$

20 $$w_{j}^{sm} = \frac{1}{d}\mathop \sum \limits_{k = 1}^{d} w_{jk}^{s}$$

21 $$w_{j}^{su} = \mathop {\max }\limits_{k} w_{jk}^{s}$$

*Step 7.* Determine the distribution parameters of objective weights. The results of Step 4 are used to define a random variable for objective weights ($w_{j}^{o}$), which follows the triangular distribution. The lower ($w_{j}^{ol}$), middle ($w_{j}^{om}$), and upper ($w_{j}^{ou}$) parameters of this random variable can be calculated as follows.

22 $$w_{j}^{ol} = \mathop {\min }\limits_{k} w_{jk}^{o}$$

23 $$w_{j}^{om} = \frac{1}{d}\mathop \sum \limits_{k = 1}^{d} w_{jk}^{o}$$

24 $$w_{j}^{ou} = \mathop {\max }\limits_{k} w_{jk}^{o}$$

*Step 8.* Compute the distribution parameters of combined weights. In this step, a random variable is defined to obtain a combination of the subjective and objective weights of criteria. Let $w_{j}^{c}$ denote the random variable for the combined weights of criteria. The following equations are used to compute the lower ($w_{j}^{cl}$), middle ($w_{j}^{cm}$), and upper ($w_{j}^{cu}$) parameters of

25 $$w_{j}^{cl} = \frac{1}{2}\left( {w_{j}^{sl} + w_{j}^{ol} } \right)$$

26 $$w_{j}^{cm} = \frac{1}{2}\left( {w_{j}^{sm} + w_{j}^{om} } \right)$$

27 $$w_{j}^{cu} = \frac{1}{2}\left( {w_{j}^{su} + w_{j}^{ou} } \right)$$

*Step 9.* Start the Monte Carlo simulation. In this step, we should define the number iterations ($N_{itr}$) and set the iteration counter to 1 ($itr = 1$). Moreover, a new variable is defined which shows the score of $i$ th alternative at the $r$ th place ($SC_{ir}$), and it is set to zero ($SC_{ir} = 0$) in this step.

*Step 10.* Generate random weights for the criteria. In this step, a set of weights is generated based on the triangular distribution of combined criteria weights defined in Step 8. The sum of criteria weights in an MCDM problem is needed to be equal to 1. Generating a set of random numbers may lead to numbers that do not meet this requirement. Therefore, the generated numbers are divided by their summation to be scaled.

*Step 11.* Generate a random decision matrix. Based on the parameters obtained in Step 5, a decision matrix is generated in this step.

*Step 12.* Solve the generated MCDM problem using the WASPAS method. The generated criteria weights of Step 10 and the decision matrix generated in Step 11 are used as inputs for the WASPAS method. According to the solution determined by WASPAS, the score variable will be changed in this step. If the $i$ th alternative is placed at the $r$ th rank, then increase the value of $SC_{ir}$ by one ($SC_{ir} = SC_{ir} + 1$). The iteration counter is also increased by one ($itr = itr + 1$).

*Step 13.* If the iteration counter is less than or equal to the defined value ($itr \le N_{itr}$) go to Step 10 (do the iteration again). If it’s not, calculate the normalized score values ($NSC_{ir}$) using the following equation.

28 $$NSC_{ir} = \frac{{SC_{ir} }}{{N_{itr} }}$$

The values of $NSC_{ir}$ are in the range of 0 to 1.

*Step 14.* Solve the assignment model. The assignment model defined in Eq. (15) is used in this step to determine the final rank of the alternatives. In Eq. (15), $\pi_{ir}$ are replaced with $NSC_{ir}$ as the measure of concordance among the criteria on ranking the $i$ th alternative at the $r$ th place. Solving this model yields the rank of each alternative, and we can assess each alternative based on its rank.

## Results and discussion

In this section, the proposed approach is applied to a case of distribution center location assessment problem. Firstly, the characteristics of the problem are described. Then the steps of the proposed are used to deal with the problem. A comparative analysis is finally performed to verify the validity of the results.

### Problem description

**Figure 3:**
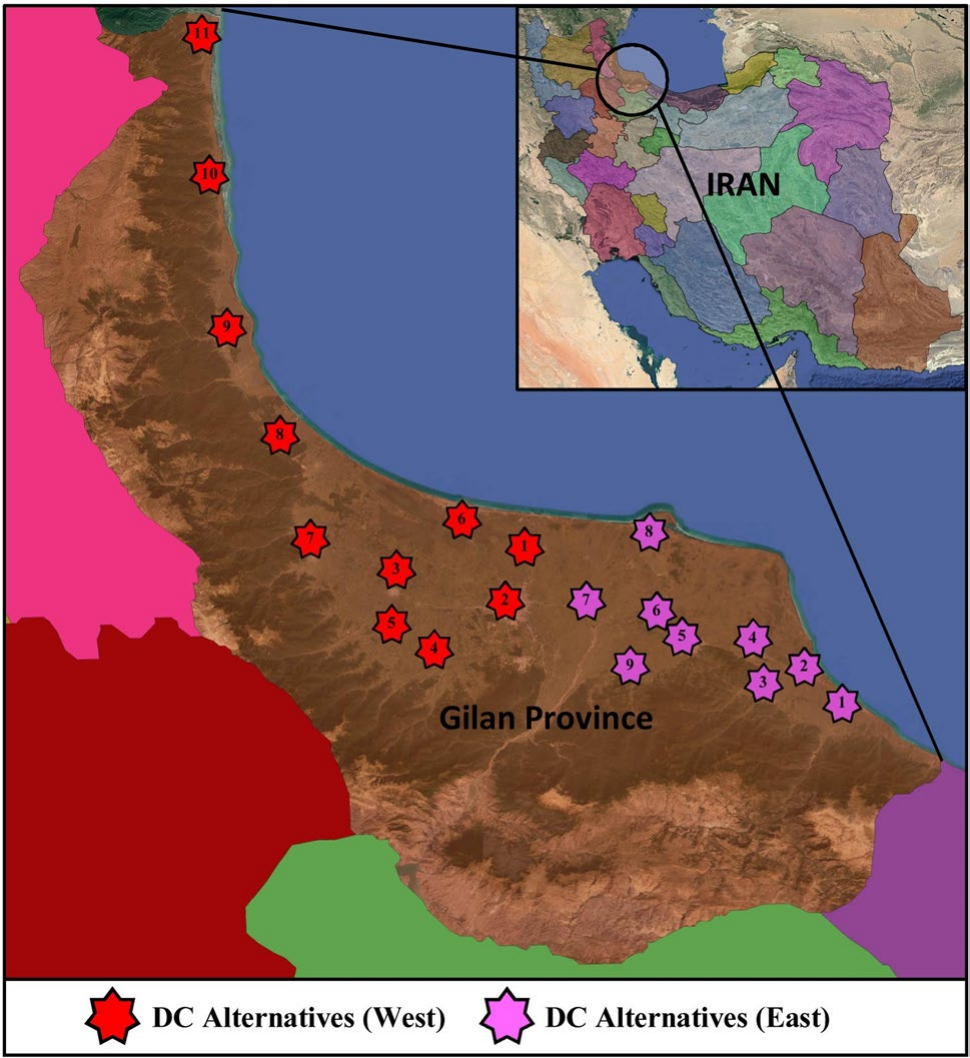
The geographical location of the alternatives (created using Google Earth Pro 7, https://www.google.com/earth).

The problem presented in this section is related to the assessment of several locations to establish some distribution centers for a company in Iran. The company, founded in the 1960s, produces a range of detergent and hygienic products, and it has widely been expanded in the past decades. This company has a new plan to extend its logistics network by establishing new distribution centers in the different provinces of Iran. Gilan Province is one of the targets of establishing new distribution centers. Dividing the province into two zones (the west and east zones), the company needs to establish two distribution centers in this province. One of the centers is to serve the west zone, and the other should serve the east zone. The west zone includes eleven potential cities, which can be considered as possible alternatives for establishing new distribution centers, and the east zone comprises nine potential cities to be assessed. The list of cities or possible alternatives in each zone is presented in Table 4, and Fig. 3 shows the geographical location of these cities.

**Table 4:** The list of possible alternatives.

West zone		East zone	
Alternative	City	Alternative	City
$A_{1}$(West)	Khomam	$A_{1}$(East)	Kelachay
$A_{2}$(West)	Rasht	$A_{2}$(East)	Rudsar
$A_{3}$(West)	Someh Sara	$A_{3}$(East)	Amlash
$A_{4}$(West)	Shaft	$A_{4}$(East)	Langarud
$A_{5}$(West)	Fuman	$A_{5}$(East)	Lahijan
$A_{6}$(West)	Bandar Anzali	$A_{6}$(East)	Astaneh-ye Ashrafiyeh
$A_{7}$(West)	Masal	$A_{7}$(East)	Kuchesfahan
$A_{8}$(West)	Rezvanshahr	$A_{8}$(East)	Kiashahr
$A_{9}$(West)	Talesh	$A_{9}$(East)	Siahkal
$A_{10}$(West)	Haviq		
$A_{11}$(West)	Astara

The board of directors of the company formed a group of ten experts from marketing, research and development (R&D), production, and finance departments to assess the possible alternatives. Table 5 shows the list of the group of experts (decision-makers) and their corresponding departments and job titles.

**Table 5:** The information about the experts.

Expert	Department	Job title	Years of experience	Gender	Academic degree
$D_{1}$	Marketing department	Chief marketing officer	12	Male	PhD in Management
$D_{2}$	Marketing department	Sales manager	6	Female	MA in Marketing
$D_{3}$	Marketing department	Promotions manager	6	Female	MA in Marketing
$D_{4}$	Marketing department	Marketing specialist	3	Female	MA in Marketing
$D_{5}$	R&D department	Project manager	10	Male	MS in Industrial Engineering
$D_{6}$	R&D department	Senior researcher	8	Male	MS in Industrial Engineering
$D_{7}$	Production department	Warehouse manager	10	Male	BA in Industrial Engineering
$D_{8}$	Production department	Operations manager	12	Male	BA in Industrial Engineering
$D_{9}$	Finance department	Finance manager	12	Male	MA in Accounting & Finance
$D_{10}$	Finance department	Risk analyst	6	Female	MA in Accounting & Finance

**Figure 4:**
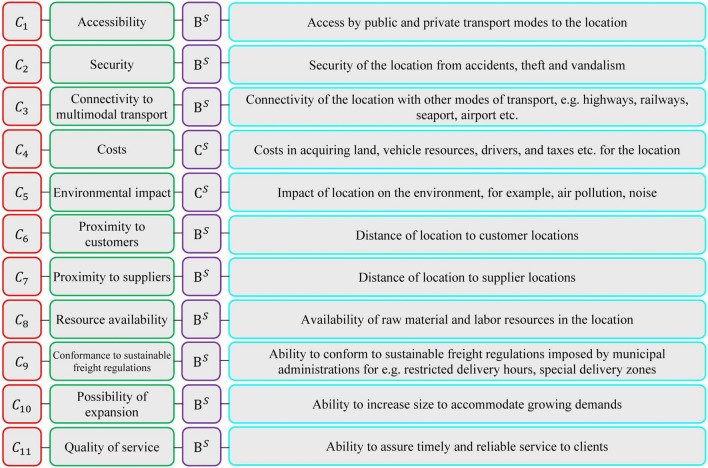
The criteria and their definitions for the assessment process.

The group of experts agreed on eleven criteria with regard to the literature and the characteristics of the problem^75^. The list of criteria, their definitions, and their types (beneficial or non-beneficial) are shown in Fig. 4.

### Application of the proposed approach

To assess the given possible locations to establish new distribution centers, we can use the proposed approach. According to the previous section, the following steps delineate the application of the proposed approach.

*Step 1.* In this step, we need to define the problem. Based on the problem description, we have eleven alternatives in the west zone, nine alternatives in the east zone, ten experts or decision-makers, and eleven criteria for the assessment process.

*Step 2.* The experts evaluated the criteria and expressed their opinion by linguistic variables defined in Table 2 of the previous section. The data relating to this evaluation is available online as supplementary material (Data Set 1)^76^. Using the SWARA II and the experts’ opinions, the criteria weights are calculated. As an example, the calculations concerned with the first expert ($D_{1}$) are shown in Table 6. Subsequently, Table 7 represents the weight of each criterion corresponding to each expert.

**Table 6:** Calculations of subjective weights for $D_{1}$.

Sorted criteria ($C_{j}$)	$t_{j}$	$RP$	$P_{{\left[ {t_{j} } \right]}}$	$PD_{{\left[ {t_{j} } \right]}}$	$V_{{\left[ {t_{j} } \right]}}$	$w_{j}^{s}$
$C_{4}$	1	L	3	0.09	2.999	0.142
$C_{1}$	2	ML	4	0.16	2.751	0.130
$C_{6}$	3	VVL	1	0.01	2.372	0.112
$C_{3}$	4	VL	2	0.04	2.348	0.111
$C_{7}$	5	VVL	1	0.01	2.258	0.107
$C_{8}$	6	MH	6	0.36	2.235	0.106
$C_{11}$	7	M	5	0.25	1.644	0.078
$C_{2}$	8	ML	4	0.16	1.315	0.062
$C_{10}$	9	VL	2	0.04	1.134	0.054
$C_{5}$	10	L	3	0.09	1.090	0.052
$C_{9}$	11	–	–	–	1	0.047

**Table 7:** The subjective weights of criteria related to each expert.

	$D_{1}$	$D_{2}$	$D_{3}$	$D_{4}$	$D_{5}$	$D_{6}$	$D_{7}$	$D_{8}$	$D_{9}$	$D_{10}$
$w_{1k}^{s}$	0.130	0.117	0.126	0.104	0.112	0.090	0.116	0.111	0.112	0.090
$w_{2k}^{s}$	0.062	0.078	0.063	0.054	0.066	0.060	0.053	0.051	0.061	0.059
$w_{3k}^{s}$	0.111	0.128	0.109	0.090	0.102	0.113	0.100	0.084	0.089	0.086
$w_{4k}^{s}$	0.142	0.139	0.134	0.195	0.129	0.133	0.141	0.129	0.152	0.203
$w_{5k}^{s}$	0.052	0.061	0.061	0.073	0.052	0.061	0.042	0.081	0.081	0.059
$w_{6k}^{s}$	0.112	0.097	0.132	0.131	0.094	0.118	0.122	0.110	0.103	0.124
$w_{7k}^{s}$	0.107	0.101	0.127	0.078	0.162	0.128	0.121	0.141	0.107	0.122
$w_{8k}^{s}$	0.106	0.077	0.080	0.074	0.086	0.090	0.098	0.088	0.088	0.087
$w_{9k}^{s}$	0.047	0.063	0.058	0.056	0.060	0.058	0.037	0.065	0.053	0.049
$w_{10k}^{s}$	0.054	0.074	0.064	0.075	0.068	0.077	0.072	0.052	0.077	0.054
$w_{11k}^{s}$	0.078	0.064	0.047	0.070	0.069	0.071	0.099	0.087	0.078	0.069

*Step 3.* In this step, ten decision-matrices are defined based on the experts’ opinions on the rating of each alternative in the west and east zones concerning each criterion. The experts’ opinions are elicited using the linguistic variables presented in Table 2 and transformed into numerical values to construct each decision matrix. To clarify the procedure, the evaluations of $D_{1}$ for the east zone and the corresponding decision matrix are shown in Table 8 as an example. The data of this step is also available online as supplementary material (Data Set 2)^76^.

**Table 8:** The evaluations of $D_{1}$ for the east zone.

		$C_{1}$	$C_{2}$	$C_{3}$	$C_{4}$	$C_{5}$	$C_{6}$	$C_{7}$	$C_{8}$	$C_{9}$	$C_{10}$	$C_{11}$
Evaluation based on linguistic variables	$A_{1}$(East)	L	MH	L	H	MH	ML	L	M	ML	ML	L
	$A_{2}$(East)	L	ML	M	M	ML	M	ML	L	H	ML	MH
	$A_{3}$(East)	M	H	MH	M	MH	MH	M	MH	MH	M	M
	$A_{4}$(East)	MH	M	H	MH	ML	MH	M	MH	M	MH	MH
	$A_{5}$(East)	H	VH	M	H	ML	H	ML	H	H	M	VH
	$A_{6}$(East)	M	MH	MH	M	L	MH	H	MH	ML	MH	ML
	$A_{7}$(East)	H	MH	MH	H	M	M	M	M	H	H	MH
	$A_{8}$(East)	M	M	M	MH	H	M	L	M	ML	ML	M
	$A_{9}$(East)	MH	M	M	MH	M	ML	ML	ML	ML	M	ML
Numerical decision matrix	$A_{1}$(East)	3	6	3	7	6	4	3	5	4	4	3
	$A_{2}$(East)	3	4	5	5	4	5	4	3	7	4	6
	$A_{3}$(East)	5	7	6	5	6	6	5	6	6	5	5
	$A_{4}$(East)	6	5	7	6	4	6	5	6	5	6	6
	$A_{5}$(East)	7	8	5	7	4	7	4	7	7	5	8
	$A_{6}$(East)	5	6	6	5	3	6	7	6	4	6	4
	$A_{7}$(East)	7	6	6	7	5	5	5	5	7	7	6
	$A_{8}$(East)	5	5	5	6	7	5	3	5	4	4	5
	$A_{9}$(East)	6	5	5	6	5	4	4	4	4	5	4

*Step 4.* Based on the decision-matrices constructed in the previous step, the MEREC method is used to determine ten sets of the objective criteria weights for each zone so that each of these sets is related to one of the decision-matrices. The results are presented in Table 9.

**Table 9:** The objective criteria weights for each zone.

Zone	Experts ($D_{k}$)	$w_{1k}^{o}$	$w_{2k}^{o}$	$w_{3k}^{o}$	$w_{4k}^{o}$	$w_{5k}^{o}$	$w_{6k}^{o}$	$w_{7k}^{o}$	$w_{8k}^{o}$	$w_{9k}^{o}$	$w_{10k}^{o}$	$w_{11k}^{o}$
West zone	$D_{1}$	0.083	0.066	0.083	0.074	0.140	0.115	0.096	0.117	0.075	0.073	0.078
	$D_{2}$	0.104	0.104	0.051	0.095	0.090	0.089	0.119	0.145	0.074	0.063	0.066
	$D_{3}$	0.109	0.093	0.102	0.107	0.091	0.084	0.116	0.148	0.057	0.042	0.051
	$D_{4}$	0.120	0.091	0.102	0.070	0.080	0.087	0.116	0.092	0.058	0.117	0.066
	$D_{5}$	0.110	0.044	0.101	0.061	0.089	0.096	0.225	0.048	0.089	0.048	0.088
	$D_{6}$	0.072	0.055	0.101	0.068	0.082	0.114	0.144	0.161	0.067	0.078	0.056
	$D_{7}$	0.128	0.110	0.088	0.100	0.066	0.090	0.139	0.091	0.075	0.037	0.076
	$D_{8}$	0.100	0.050	0.064	0.084	0.132	0.086	0.126	0.102	0.043	0.139	0.074
	$D_{9}$	0.142	0.093	0.069	0.071	0.094	0.076	0.141	0.089	0.072	0.078	0.074
	$D_{10}$	0.106	0.095	0.118	0.076	0.063	0.041	0.124	0.142	0.068	0.106	0.061
East zone	$D_{1}$	0.124	0.086	0.135	0.040	0.095	0.064	0.086	0.130	0.061	0.053	0.126
	$D_{2}$	0.085	0.132	0.113	0.053	0.066	0.106	0.181	0.096	0.070	0.045	0.053
	$D_{3}$	0.067	0.136	0.123	0.043	0.023	0.044	0.082	0.110	0.123	0.135	0.112
	$D_{4}$	0.058	0.097	0.115	0.022	0.080	0.110	0.180	0.109	0.063	0.043	0.124
	$D_{5}$	0.069	0.079	0.102	0.046	0.087	0.083	0.163	0.131	0.063	0.032	0.145
	$D_{6}$	0.121	0.075	0.119	0.059	0.064	0.070	0.147	0.069	0.054	0.106	0.115
	$D_{7}$	0.143	0.097	0.073	0.039	0.061	0.135	0.100	0.120	0.133	0.057	0.041
	$D_{8}$	0.054	0.080	0.128	0.019	0.067	0.103	0.088	0.137	0.093	0.107	0.125
	$D_{9}$	0.082	0.084	0.121	0.040	0.019	0.060	0.097	0.135	0.123	0.121	0.119
	$D_{10}$	0.126	0.090	0.093	0.020	0.063	0.130	0.156	0.121	0.021	0.125	0.056

*Step 5.* The distribution parameters of the decision matrices are calculated in this step. Based on Eqs. (16) to (18), and the matrices of Step 3, we can calculate these values. The parameters of the triangular distributions are shown as a triplet [$x_{ij}^{l}$,$x_{ij}^{m}$,$x_{ij}^{u}$] for each zone in Tables 10 and 11.

**Table 10:** The distribution parameters of the west zone decision matrix.

	$C_{1}$	$C_{2}$	$C_{3}$	$C_{4}$	$C_{5}$	$C_{6}$	$C_{7}$	$C_{8}$	$C_{9}$	$C_{10}$	$C_{11}$
	[3,4,5]	[4,5.1,6]	[5,6.5,7]	[4,5.4,6]	[3,4.2,5]	[5,5.7,7]	[5,6.3,7]	[5,6,7]	[4,5.1,6]	[5,5.9,7]	[4,4.9,6]
$A_{1}$(West)	[8,8.5,9]	[8,8.7,9]	[8,8.4,9]	[8,8.3,9]	[3,3.5,4]	[6,7.4,8]	[8,8.3,9]	[8,8.8,9]	[5,5.8,7]	[4,5.2,6]	[6,6.7,8]
$A_{2}$(West)	[5,5.6,7]	[6,7.1,8]	[4,4.7,6]	[5,6.2,7]	[3,4.3,5]	[5,6.1,7]	[5,6.1,7]	[6,7.1,8]	[5,5.7,7]	[5,5.9,7]	[5,6,7]
$A_{3}$(West)	[5,5.9,7]	[5,6.4,7]	[4,5.3,6]	[4,4.9,6]	[4,5,6]	[5,6.4,7]	[5,6.1,7]	[5,5.7,7]	[4,4.5,6]	[5,5.9,7]	[4,5.4,6]
$A_{4}$(West)	[5,6.2,7]	[6,7,8]	[4,5.3,6]	[6,6.7,7]	[4,4.7,6]	[5,6.3,7]	[5,5.6,7]	[6,7,8]	[5,6.3,7]	[5,5.9,7]	[5,5.9,7]
$A_{5}$(West)	[5,6,7]	[6,7.2,8]	[5,5.9,7]	[6,7,8]	[3,4,5]	[5,6.1,7]	[6,6.7,8]	[6,6.8,8]	[5,6.2,7]	[4,5.1,6]	[5,5.7,7]
$A_{6}$(West)	[3,3.9,5]	[4,4.9,6]	[3,3.7,5]	[4,4.6,6]	[5,5.8,7]	[6,7.4,8]	[4,5.1,6]	[3,3.8,5]	[4,4.9,6]	[3,4,5]	[4,5.1,6]
$A_{7}$(West)	[4,5.1,6]	[5,6,7]	[3,4.1,5]	[5,6,7]	[2,3,4]	[5,6,7]	[3,4.1,5]	[4,5.3,6]	[4,5.1,6]	[4,5.4,6]	[4,5.3,6]
$A_{8}$(West)	[3,3.9,5]	[5,5.8,7]	[3,4.1,5]	[5,5.5,6]	[3,4.6,5]	[4,5.3,6]	[3,4.1,5]	[4,5.4,6]	[4,5.3,6]	[4,5.3,6]	[4,5.5,6]
$A_{9}$(West)	[3,3.9,5]	[5,5.9,7]	[3,4,5]	[5,5.7,6]	[2,3.1,4]	[4,4.9,6]	[2,3.3,4]	[5,6,7]	[5,6,7]	[4,5,6]	[4,5.2,6]
$A_{10}$(West)	[4,5.2,6]	[5,5.8,7]	[4,4.7,6]	[5,6.4,7]	[3,4,5]	[4,4.8,6]	[4,4.8,6]	[5,5.6,7]	[5,6.1,7]	[4,5.1,6]	[4,4.4,5]

**Table 11:** The distribution parameters of the east zone decision matrix.

	$C_{1}$	$C_{2}$	$C_{3}$	$C_{4}$	$C_{5}$	$C_{6}$	$C_{7}$	$C_{8}$	$C_{9}$	$C_{10}$	$C_{11}$
$A_{1}$(East)	[3,3.9,5]	[4,5.7,6]	[3,3.5,5]	[5,6.5,7]	[4,5.1,6]	[3,4.2,5]	[2,2.9,4]	[3,4,5]	[3,4,5]	[3,3.8,5]	[3,4.3,5]
$A_{2}$(East)	[3,3.9,5]	[4,4.4,6]	[3,4.4,5]	[5,6.6,7]	[4,4.8,6]	[4,4.9,6]	[2,3.1,4]	[3,3.8,5]	[5,6.3,7]	[4,5.1,6]	[5,5.7,6]
$A_{3}$(East)	[5,6.1,7]	[5,6.4,7]	[4,5.1,6]	[5,6.2,7]	[4,5.3,6]	[5,6,7]	[3,3.8,5]	[5,5.9,7]	[4,4.6,6]	[4,5.1,6]	[4,5,6]
$A_{4}$(East)	[5,5.9,7]	[5,6.3,7]	[6,6.6,7]	[5,6.4,7]	[4,4.7,6]	[5,5.7,7]	[3,4.5,5]	[5,5.7,7]	[5,6,7]	[4,5.1,6]	[5,6.3,7]
$A_{5}$(East)	[6,7.3,8]	[8,8.2,9]	[5,5.7,7]	[6,7,8]	[3,4.1,5]	[5,5.8,7]	[4,4.5,6]	[5,5.9,7]	[5,6,7]	[5,5.5,7]	[7,7.4,8]
$A_{6}$(East)	[5,5.8,7]	[5,6.1,7]	[5,5.2,6]	[5,6.1,7]	[3,3.8,5]	[4,5,6]	[5,5.8,7]	[5,6.2,7]	[4,4.9,6]	[5,6.5,7]	[4,4.8,6]
$A_{7}$(East)	[5,6,7]	[5,6.1,7]	[6,7,8]	[5,5.7,7]	[3,4.2,5]	[3,4.4,5]	[5,6.3,7]	[5,6,7]	[5,6.1,7]	[5,6.4,7]	[4,5.3,6]
$A_{8}$(East)	[4,5.1,6]	[4,5.1,6]	[4,5.1,6]	[4,5.6,6]	[5,5.8,7]	[4,5.3,6]	[3,4.1,5]	[3,4,5]	[3,4.1,5]	[3,4.3,5]	[3,3.9,5]
$A_{9}$(East)	[4,5.1,6]	[3,4.2,5]	[3,4,5]	[5,5.6,6]	[5,5.8,7]	[4,5.1,6]	[4,4.9,6]	[4,5.3,6]	[4,5.2,6]	[4,5,6]	[3,4.1,5]

*Steps 6 to 8.* According to the results of Step 2 and Eqs. (19) to (21), the parameters of the triangular distribution for the subjective weights are determined. These parameters are shown in Table 12 in the form of [$w_{j}^{sl}$,$w_{j}^{sm}$,$w_{j}^{su}$]. Moreover, the parameters of objective criteria weights related to each zone are calculated based on the results of Step 4 and Eqs. (22) to (24). Subsequently, the combined weights are determined using Eqs. (25) to (27). The triplets connected with the objective weights [$w_{j}^{ol}$,$w_{j}^{om}$,$w_{j}^{ou}$] and combined weights [$w_{j}^{cl}$,$w_{j}^{cm}$,$w_{j}^{cu}$] for each zone are also represented in Table 12.

**Table 12:** The distribution parameters of the criteria weights.

Criteria	$w_{j}^{s}$	$w_{j}^{o}$		$w_{j}^{c}$	
		West zone	East zone	West zone	East zone
$C_{1}$	[0.09,0.111,0.13]	[0.072,0.107,0.142]	[0.054,0.093,0.143]	[0.081,0.109,0.136]	[0.072,0.102,0.137]
$C_{2}$	[0.051,0.061,0.078]	[0.044,0.08,0.11]	[0.075,0.096,0.136]	[0.048,0.071,0.094]	[0.063,0.078,0.107]
$C_{3}$	[0.084,0.101,0.128]	[0.051,0.088,0.118]	[0.073,0.112,0.135]	[0.068,0.095,0.123]	[0.078,0.107,0.131]
$C_{4}$	[0.129,0.15,0.203]	[0.061,0.081,0.107]	[0.019,0.038,0.059]	[0.095,0.115,0.155]	[0.074,0.094,0.131]
$C_{5}$	[0.042,0.062,0.081]	[0.063,0.093,0.14]	[0.019,0.062,0.095]	[0.053,0.077,0.11]	[0.031,0.062,0.088]
$C_{6}$	[0.094,0.114,0.132]	[0.041,0.088,0.115]	[0.044,0.091,0.135]	[0.067,0.101,0.124]	[0.069,0.102,0.134]
$C_{7}$	[0.078,0.119,0.162]	[0.096,0.135,0.225]	[0.082,0.128,0.181]	[0.087,0.127,0.193]	[0.08,0.124,0.172]
$C_{8}$	[0.074,0.087,0.106]	[0.048,0.114,0.161]	[0.069,0.116,0.137]	[0.061,0.1,0.133]	[0.072,0.102,0.121]
$C_{9}$	[0.037,0.055,0.065]	[0.043,0.068,0.089]	[0.021,0.08,0.133]	[0.04,0.061,0.077]	[0.029,0.067,0.099]
$C_{10}$	[0.052,0.067,0.077]	[0.037,0.078,0.139]	[0.032,0.083,0.135]	[0.044,0.072,0.108]	[0.042,0.075,0.106]
$C_{11}$	[0.047,0.073,0.099]	[0.051,0.069,0.088]	[0.041,0.102,0.145]	[0.049,0.071,0.093]	[0.044,0.087,0.122]

**Figure 5:**
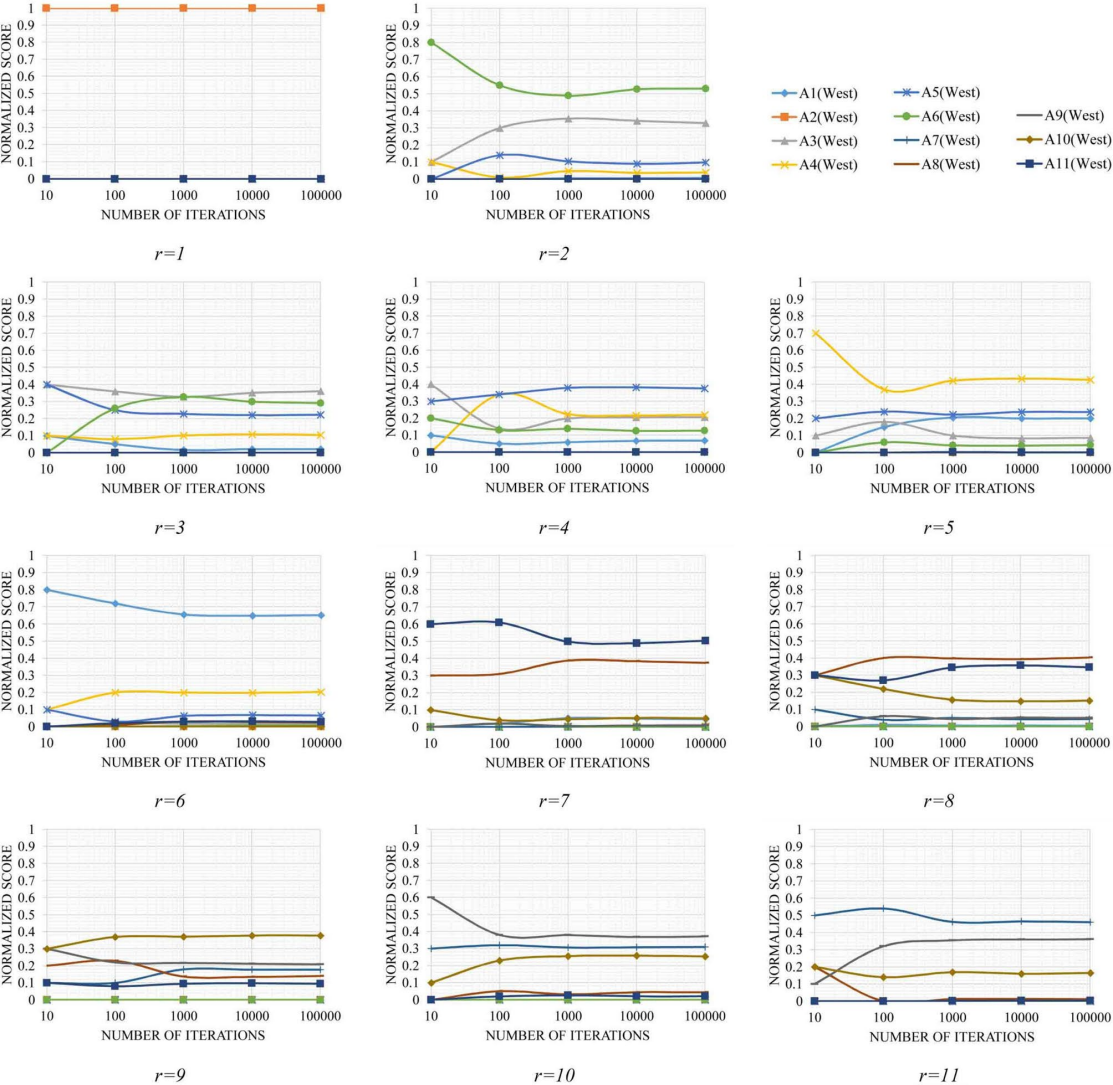
Variations in $NSC_{ir}$ in the different number of iterations for the west zone.

**Figure 6:**
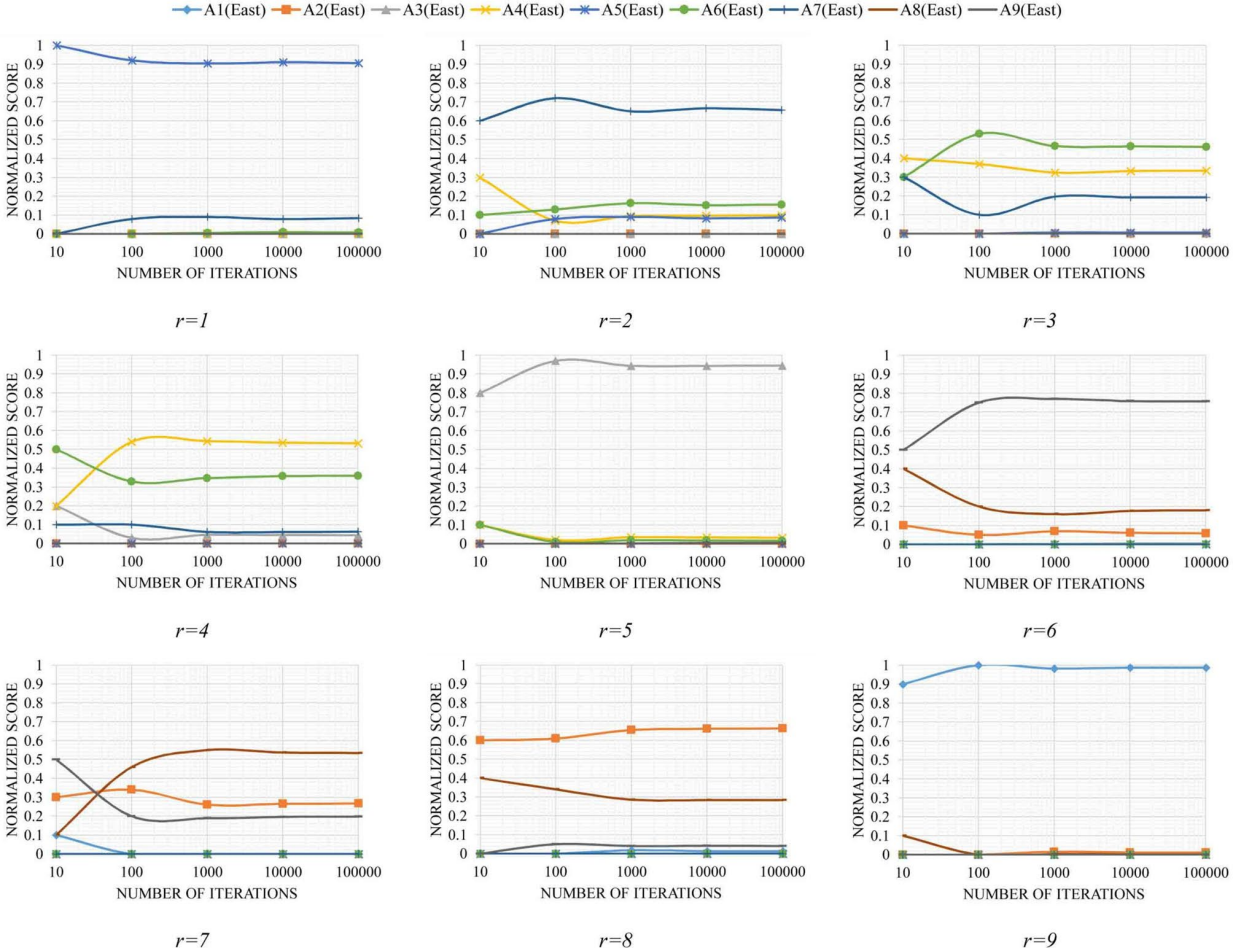
Variations in $NSC_{ir}$ in the different number of iterations for the east zone.

*Steps 9 to 13.* In these steps, the Monte Carlo simulation is used to assess the possible distribution centers in each zone. To obtain the normalized score values, the simulation was performed by defining different values for the number of iterations or $N_{itr}$ (10, 100, 1000, 10,000, and 100,000). The detailed results of these steps are available online as supplementary material (Data Set 3)^76^. However, the values of $NSC_{ir}$ for the west and east zones based on different values of $N_{itr}$ are schematically presented in Figs. 5 and 6. Since the values of $N_{itr}$ were exponentially increased, a logarithmic scale is used for the related axis to present these diagrams.

According to Figs. 5 and 6, a convergence of the values of $NSC_{ir}$ can be seen in different ranks when the number of iterations increases. The convergence of these random variables is very important since we can expect that these sequences eventually take constant values.

*Step 14.* Based on the values of $NSC_{ir}$ calculated in the previous step and the assignment model described in Eq. (15), we can rank and assess the possible alternatives for establishing new distribution centers. Because of the convergence of the values of $NSC_{ir}$, the values related to the greatest number of iterations ($N_{itr}$ = 100,000), as shown in Tables 13 and 14, are used in this step to rank the possible alternatives in each zone. The objective function of Eq. (15) is replaced with the following equation to obtain the final rank.

29 $$Max\;f = \mathop \sum \limits_{i = 1}^{n} \mathop \sum \limits_{r = 1}^{n} NSC_{ir} s_{ir}$$

**Table 13:** The values of $NSC_{ir}$ for the west zone.

*i*	*r*										
	1	2	3	4	5	6	7	8	9	10	11
1	0	0.0060	0.0220	0.0680	0.2010	0.6520	0.0451	0.0056	0	0	0
2	1	0	0	0	0	0	0	0	0	0	0
3	0	0.3279	0.3604	0.2067	0.0872	0.0177	0.0001	0	0	0	0
4	0	0.0387	0.1046	0.2205	0.4266	0.2036	0.0057	0.0002	0	0	0
5	0	0.0973	0.2223	0.3769	0.2375	0.0654	0.0006	0	0	0	0
6	0	0.5302	0.2907	0.1277	0.0447	0.0067	0	0	0	0	0
7	0	0	0	0	0	0	0.0087	0.0436	0.1774	0.3095	0.4607
8	0	0	0	0.0001	0.0016	0.0247	0.3744	0.4034	0.1401	0.0440	0.0118
9	0	0	0	0	0	0	0.0098	0.0499	0.2080	0.3719	0.3604
10	0	0	0	0	0	0.0012	0.0514	0.1510	0.3787	0.2540	0.1637
11	0	0	0	0	0.0014	0.0286	0.5042	0.3464	0.0955	0.0206	0.0033

**Table 14:** The values of $NSC_{ir}$ for the east zone.

*i*	*r*								
	1	2	3	4	5	6	7	8	9
1	0	0	0	0	0	0	0.0004	0.0125	0.9871
2	0	0	0	0	0	0.0585	0.2668	0.6638	0.0109
3	0	0.0004	0.0047	0.0442	0.9454	0.0052	0.0001	0	0
4	0.0024	0.0987	0.3344	0.5325	0.0321	0	0	0	0
5	0.9059	0.0875	0.0061	0.0005	0	0	0	0	0
6	0.0070	0.1560	0.4614	0.3599	0.0157	0	0	0	0
7	0.0847	0.6575	0.1934	0.0630	0.0015	0	0	0	0
8	0	0	0	0	0.0003	0.1800	0.5347	0.2831	0.0020
9	0	0	0	0	0.0050	0.7563	0.1981	0.0407	0.0001

Solving the assignment model yields the ranking presented in Table 15. It should be noted that the simulation process was performed by MATLAB R2014a, and the assignment model was solved using LINGO Extended × 64. All computations were carried out on a PC with a 2.4 GHz CPU (Intel® Core™i5-520 M), 8 gigabytes of RAM, and Windows 8.1 Pro (64 bit) operating system.

**Table 15:** The final ranking of the alternatives.

West zone		East zone	
Alternative	Rank	Alternative	Rank
$A_{1}$(West)	6	$A_{1}$(East)	9
$A_{2}$(West)	1	$A_{2}$(East)	8
$A_{3}$(West)	3	$A_{3}$(East)	5
$A_{4}$(West)	5	$A_{4}$(East)	4
$A_{5}$(West)	4	$A_{5}$(East)	1
$A_{6}$(West)	2	$A_{6}$(East)	3
$A_{7}$(West)	11	$A_{7}$(East)	2
$A_{8}$(West)	8	$A_{8}$(East)	7
$A_{9}$(West)	10	$A_{9}$(East)	6
$A_{10}$(West)	9		
$A_{11}$(West)	7		

The results show that $A_{2}$ has been ranked first in the west zone, so we can say that Rasht is the best city to establish a new distribution center for the company. Moreover, we can see that $A_{6}$ and $A_{3}$ are in the second and third places of the ranking, respectively. Rasht is the largest and capital city of Gilan Province, and it has developed more than the other cities of Gilan. Opening new distribution centers in this city seems completely rational because of the high level of infrastructure and resources. However, the costs of establishing and handling a new distribution center may be very high in Rasht. A large part of the costs is related to the cost of buying land, which is much lower for the other cities. As a result, Bandar Anzali ($A_{6}$) and Someh Sara ($A_{3}$) can also be considered by the company as viable alternatives to Rasht. In the east zone, $A_{5}$ has been ranked first, and therefore Lahijan can be considered as the best city to open a new distribution center for the company. This city is also one of the costly cities of Gilan Province, so considering additional options could be helpful for decision-makers. As can be seen in Table 15, $A_{7}$ and $A_{6}$ have been ranked second and third. Consequently, Kuchesfahan ($A_{7}$) and Astaneh-ye Ashrafiyeh ($A_{6}$) can be acceptable substitutes for Lahijan to establish a new distribution center. In fact, the assessment has been made based on limited information and experts’ opinion. If there is additional information, the company might need to make a further assessment to establish new distribution centers. Although additional information could change the best choice for each zone, the obtained ranking can be seen as a basis for the final assessment.

To verify the results of the proposed approach, the middle parameters of the decision-matrices ($x_{ij}^{m}$) related to each zone, along with equal criteria weights, have been used as inputs for some existing MCDM methods. The results of Simple Additive Weighting (SAW), Complex Proportional Assessment (COPRAS), TOPSIS, and Evaluation based on Distance from Average Solution (EDAS) are summarized in Table 16. In this table, Spearman's correlations ($rho$) between the results of the proposed approach and those of the other methods are also presented. As can be seen in Table 16, all the correlation values are greater than 0.8, which shows a very strong relationship^77^, so it can be concluded that the proposed approach yields results that are congruent with the results of other MCDM methods. Since the proposed approach takes the spectrum of experts’ opinions through triangular distribution, its results are more reliable than the results obtained just based upon the middle parameters.

**Table 16:** Results of the comparative analysis.

	Alternative	Proposed approach	SAW	COPRAS	TOPSIS	EDAS
West zone	$A_{1}$	6	6	6	6	6
	$A_{2}$	1	1	1	1	1
	$A_{3}$	3	4	4	4	4
	$A_{4}$	5	5	5	5	5
	$A_{5}$	4	3	3	3	3
	$A_{6}$	2	2	2	2	2
	$A_{7}$	11	11	11	10	11
	$A_{8}$	8	7	7	7	7
	$A_{9}$	10	10	10	11	10
	$A_{10}$	9	8	9	8	9
	$A_{11}$	7	9	8	9	8
	$rho$	–	0.964	0.982	0.955	0.982
East zone	$A_{1}$	9	9	9	9	9
	$A_{2}$	8	7	7	7	7
	$A_{3}$	5	5	5	5	5
	$A_{4}$	4	4	3	3	3
	$A_{5}$	1	1	1	1	1
	$A_{6}$	3	3	4	4	4
	$A_{7}$	2	2	2	2	2
	$A_{8}$	7	8	8	8	8
	$A_{9}$	6	6	6	6	6
	$rho$	–	0.983	0.967	0.967	0.967

It should be noted that the objective weighting method, subjective weighting method, and the ranking method utilized in the assessment framework of this research could be replaced with other appropriate methods. Nevertheless, the proposed approach has some advantages compared with the other approaches which are used in the assessment problems with multiple criteria. Firstly, the modified version of SWARA, which is introduced in this study, is designed so that the subjective criteria weights can be determined in a more intelligible and efficient way than the original SWARA and some other subjective weighting approaches. Secondly, SWARA II has been integrated with a newly introduced method that uses a new perspective based on the exclusion and removal effects of criteria to obtain their weights. Therefore, the proposed approach is founded on the integration of the simplicity of SWARA II and the new perspective of MEREC. Since the whole framework is novel, it cannot be compared with the existing integrated MCDM frameworks in a comprehensive way, but this research has presented a comparison of the results based on a simplification. Finally, comparing to the other multi-expert and group decision-making approaches, the proposed approach aggregates the experts’ opinions by employing a simulation-based process and the assignment model rather than using simple mathematical aggregation operators. This attribute helps to get the results that incorporate the experts’ opinions efficiently and reasonably. On the other hand, there are some disadvantages to the proposed approach. One of them is the complexity of the simulation process.

### Consent to publish

Informed consent was obtained to publish the information presented.

## Conclusion

Due to the importance of distribution centers in supply chain management and logistics, selecting appropriate locations for them becomes essential for several companies. Assessment of the locations for establishing distribution centers is usually categorized as an MCDM problem. In this study, a new MCDM method has been proposed by integration of SWARA II, MEREC, WASPAS, Monte Carlo simulation, and the assignment model. Both subjective and objective weights of criteria have been taken into account in the proposed approach, and the evaluation has been made based on a multi-expert process. An improved version of the SWARA method, called SWARA II, has also been introduced to simplify the determination of subjective criteria weights. Unlike many studies which focused on simple aggregation operators to model experts’ opinions, a Monte Carlo simulation based on the triangular distribution has been utilized in this study to model the spectrum concerned with the subjective weights, objective weights, and decision matrix elements. The proposed approach has been applied to a case of distribution center locations assessment. The problem was defined based on the need for a company to establish two distribution centers in two zones (west and east) of Gilan Province in Iran. Eleven cities in the west zone and nine cities in the east zone of the province have been assessed based on the proposed approach. The results showed that Rasht and Lahijan could be considered as best alternatives for the west and east zones, respectively. The ranking resulted from the proposed approach has been compared with the results of four other MCDM methods. The correlation values obtained demonstrate that the results of the proposed approach are valid.

## Supplementary Information

Supplementary Information 1.

Supplementary Information 2.

Supplementary Information 3.

## Data Availability

All data generated or analyzed during this study are included in this published article.
